# Nineteenth Annual Report of the New York Institution for the Blind, to the Legislature of the State

**Published:** 1855-03

**Authors:** 


*Nineteenth Annual Report of the Managers of the New York Institution for the Blind, to the Legislature of the State.*

This excellent Institution is under the superintendence of Mr. T. Colden Cooper. From the report of this gentleman for the year 1854, it appears that the whole number of blind persons receiving education, or the means of support, through the medium of the Institute, is *two hundred and five.* Of this number one hundred and forty-two are pupils; the rest are blind work people and teachers.

The physician, Dr. J. W. Clements, has recorded three deaths during the past year—all from affections of the bowels—to which class of diseases he states the blind are particularly subject.

